# Deep learning for the diagnosis of suspicious thyroid nodules based on multimodal ultrasound images

**DOI:** 10.3389/fonc.2022.1012724

**Published:** 2022-11-08

**Authors:** Yi Tao, Yanyan Yu, Tong Wu, Xiangli Xu, Quan Dai, Hanqing Kong, Lei Zhang, Weidong Yu, Xiaoping Leng, Weibao Qiu, Jiawei Tian

**Affiliations:** ^1^ Department of Ultrasound, The Second Affiliated Hospital of Harbin Medical University, Harbin, China; ^2^ The National-Regional Key Technology Engineering Laboratory for Medical Ultrasound, Guangdong Key Laboratory for Biomedical Measurements and Ultrasound Imaging, School of Biomedical Engineering, Health Science Center, Shenzhen University, Shenzhen, China; ^3^ Department of Ultrasound, The Second Hospital of Harbin, Harbin, China; ^4^ Shenzhen Key Laboratory of Ultrasound Imaging and Therapy, Paul C. Lauterbur Research Center for Biomedical Imaging, Shenzhen Institutes of Advanced Technology, Chinese Academy of Sciences, Shenzhen, China

**Keywords:** thyroid nodule, deep learning, multimodal, ultrasound, diagnosis

## Abstract

**Objectives:**

This study aimed to differentially diagnose thyroid nodules (TNs) of Thyroid Imaging Reporting and Data System (TI-RADS) 3–5 categories using a deep learning (DL) model based on multimodal ultrasound (US) images and explore its auxiliary role for radiologists with varying degrees of experience.

**Methods:**

Preoperative multimodal US images of 1,138 TNs of TI-RADS 3–5 categories were randomly divided into a training set (n = 728), a validation set (n = 182), and a test set (n = 228) in a 4:1:1.25 ratio. Grayscale US (GSU), color Doppler flow imaging (CDFI), strain elastography (SE), and region of interest mask (Mask) images were acquired in both transverse and longitudinal sections, all of which were confirmed by pathology. In this study, fivefold cross-validation was used to evaluate the performance of the proposed DL model. The diagnostic performance of the mature DL model and radiologists in the test set was compared, and whether DL could assist radiologists in improving diagnostic performance was verified. Specificity, sensitivity, accuracy, positive predictive value, negative predictive value, and area under the receiver operating characteristics curves (AUC) were obtained.

**Results:**

The AUCs of DL in the differentiation of TNs were 0.858 based on (GSU + SE), 0.909 based on (GSU + CDFI), 0.906 based on (GSU + CDFI + SE), and 0.881 based (GSU + Mask), which were superior to that of 0.825-based single GSU (*p* = 0.014, *p*< 0.001, *p*< 0.001, and *p* = 0.002, respectively). The highest AUC of 0.928 was achieved by DL based on (G + C + E + M)US, the highest specificity of 89.5% was achieved by (G + C + E)US, and the highest accuracy of 86.2% and sensitivity of 86.9% were achieved by DL based on (G + C + M)US. With DL assistance, the AUC of junior radiologists increased from 0.720 to 0.796 (*p*< 0.001), which was slightly higher than that of senior radiologists without DL assistance (0.796 vs. 0.794, *p* > 0.05). Senior radiologists with DL assistance exhibited higher accuracy and comparable AUC than that of DL based on GSU (83.4% vs. 78.9%, *p* = 0.041; 0.822 vs. 0.825, *p* = 0.512). However, the AUC of DL based on multimodal US images was significantly higher than that based on visual diagnosis by radiologists (*p*< 0.05).

**Conclusion:**

The DL models based on multimodal US images showed exceptional performance in the differential diagnosis of suspicious TNs, effectively increased the diagnostic efficacy of TN evaluations by junior radiologists, and provided an objective assessment for the clinical and surgical management phases that follow.

## 1 Introduction

Thyroid cancer has become the most common endocrine malignancy, with an increasing incidence of approximately 7%–15% annually (1, 2). Ultrasound (US) is widely used as a first-line screening tool for the clinical examination of thyroid lesions, with the advantages of no exposure to radiation, real-time dynamic imaging, and simplicity of procedure (1, 3). Multiple versions of the Thyroid Imaging Reporting and Data System (TI-RADS) have been proposed for US imaging to standardize and improve the diagnostic consistency and accuracy of thyroid lesions, and each risk stratification system has its advantages (1, 3–6).

Nevertheless, US diagnosis of thyroid nodules (TNs) is subjective to a certain extent. Various diagnostic results of US evaluation of TNs were obtained from different observers, especially less-experienced radiologists, who showed relatively lower accuracy. In previous studies, moderate variability in the interobserver agreement was found among different TI-RADS scores (7). There was fair agreement in margin, echotexture, and echogenicity (*k* = 0.34, 0.26, and 0.34, respectively) for interobserver variability (8–10). Clinically, there is a wide range of malignant risks (approximately 2%–90%) and some overlapping US features for the TNs of TI-RADS 3–5 categories; therefore, it was difficult for radiologists to accurately differentiate between benign and malignant TNs (11–13), resulting in overdiagnosis or misdiagnosis.

Fine-needle aspiration (FNA) is a relatively effective method for the preoperative diagnosis of TNs (14). The radiologists assess the malignant probability of TNs and then recommend patients for FNA or US follow-up according to TI-RADS. However, FNA is an invasive procedure with some possible complications, such as bleeding, and FNA results are also dependent on the size, composition of TNs, and skills of radiologists. Moreover, approximately 20% of the FNA results were rendered inconclusive, which led to uncertainty in the next course of clinical treatment (15–17). The development of artificial intelligence (AI) technology has shown great potential in reducing the influence of subjectivity and improving the consistency of diagnosis.

In the past two decades, machine-learning methods have been used in TN characterization, which is usually known as “radiomics” (18, 19). Radiomics can automatically extract features in the region of interest (ROI), which tends to be difficult to discern with the naked eye. It should be noted that high-throughput features extracted by radiomics from the ROI are easily affected by the segmentation strategy and imaging parameters. Deep learning (DL) is a machine-learning concept that has shown strong capability in medical image characterization and outperforms traditional machine-learning methods. With the help of artificial neural networks, DL has been widely applied to differentiate breast, thyroid, and liver lesions with good performance (20–22). However, radiologists cannot be completely replaced with AI technology. It is crucial to integrate DL methods into clinical practice; therefore, they can aid radiologists in diagnosis, evaluation, and decision-making (23). In this study, the diagnostic performances of junior and senior radiologists with and without a DL assistant were compared.

Most previous studies using DL for the diagnosis of TNs have concentrated on grayscale US (GSU) imaging. However, beyond conventional GSU, some new US technologies such as color Doppler flow imaging (CDFI), elastography, and contrast-enhanced ultrasonography are commonly used to assist in the diagnosis of GSU for TNs, which have been proven to improve the diagnostic accuracy in the clinical evaluation (13, 24, 25). This indicated that the features of blood flow and hardness also played an important role in thyroid US diagnosis. Therefore, in our study, new DL models based on multimodal US imaging were proposed to explore their application value in improving the diagnostic accuracy of suspicious thyroid lesions and the role of auxiliary diagnosis for radiologists.

## 2 Materials and methods

### 2.1 Patients

**Figure 1:**
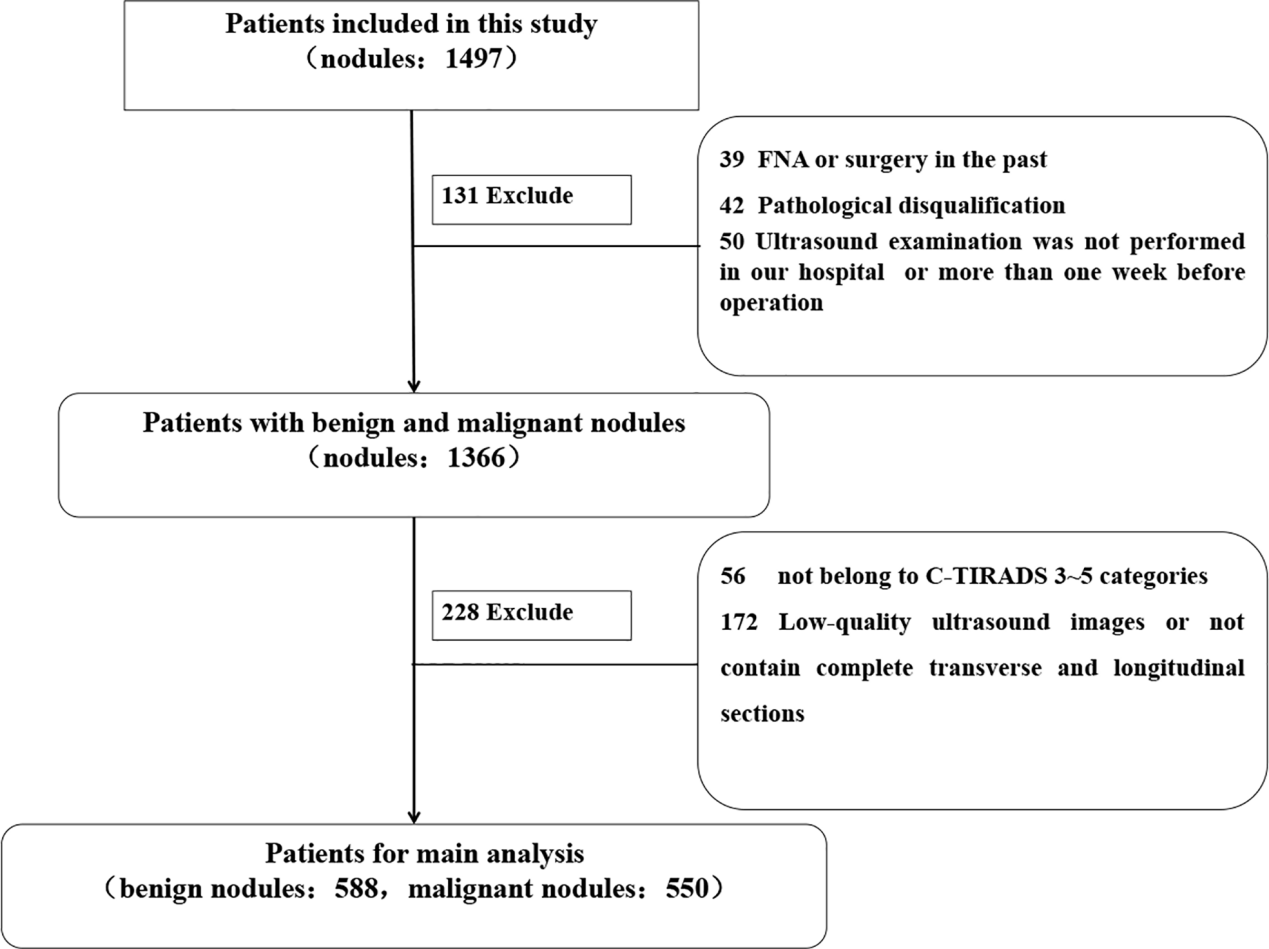
Flowchart of enrolling patients with thyroid nodules.

This retrospective study was approved by the Ethics Committee of The Second Affiliated Hospital of Harbin Medical University, and the requirement for informed consent was waived (approval number KY2021-152). Consecutive patients who had undergone thyroid surgery at The Second Affiliated Hospital of Harbin Medical University between September 9, 2020, and June 6, 2021, were enrolled. The inclusion criteria of the enrolled patients were as follows: lesions with (1) complete or high-quality transverse and longitudinal section images (2), complete surgical records and pathological results (3), no preoperative operation such as FNA and ablation or surgical treatment of TNs, and (4) US examination in our hospital within 1 week before surgery. Finally, 1,138 TNs of TI-RADS 3–5 categories from 781 patients were included in the study. The postoperative pathological results were used as the gold standard. The mean diagnostic age of patients was 47.74 ± 10.60 years (range, 21–79 years). According to the pathological results, there were 550 (48.33%) malignant and 588 (51.67%) benign TNs. The workflow of the selection is shown in **Figure 1**.

### 2.2 Ultrasound image acquisition and analysis

Preoperative thyroid US examinations were performed by two radiologists with 10 years of experience (Q.D. and H.K.) using a US device (Hitachi HI VISION Avius, Hitachi Medical Corporation, Tokyo, Japan) equipped with a 5- to 13-MHz linear probe. According to the Chinese TI-RADS (C-TIRADS) issued by the Chinese Society of Ultrasound in Medicine in 2020, thyroid scanning and imaging parameter adjustments were guided and completed (6). The GSU, CDFI, and strain elastography (SE) images of the TNs were acquired in transverse and longitudinal sections, which showed obvious characteristics and were saved in BMP format.

The ultrasonographic features were evaluated for all 1,138 TNs in our study. To maintain consistency, the images were independently analyzed by two experienced radiologists (L.Z. and W.Y.) in a double-blind manner, and results were obtained through consultation by consensus when discrepancies arose. GSU features, including the maximum diameter, position, echotexture, echogenicity, composition, orientation, margin, punctate echogenic foci, halo, and posterior features, were evaluated visually according to the C-TIRADS. CDFI could indicate tumor blood flow characteristics using the vascular distribution pattern and Adler grade (0–3) standards (26). Tumor tissue hardness was evaluated on a scale of 1–4 according to the Asteria standard by SE (27).

### 2.3 Construction of deep learning

#### 2.3.1 Pretreatment of multimodal and double-view ultrasound images

**Figure 2:**
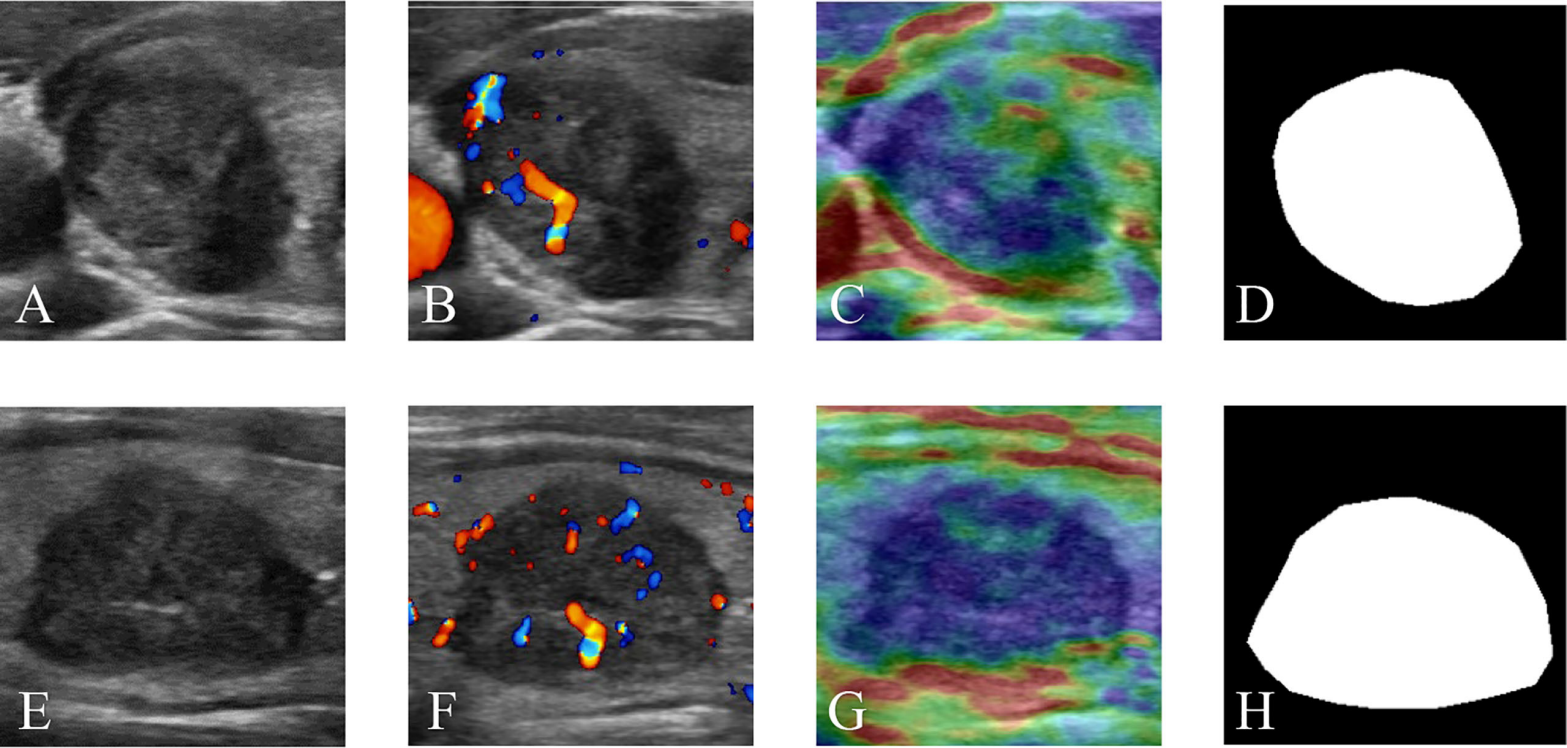
Multimodal ultrasound images of a thyroid nodule in the right lobe of a 65-year-old female patient with a pathologically proven papillary carcinoma. **(A)** GSU, **(B)** CDFI, **(C)** SE, and **(D)** Mask ultrasound images in transverse section. **(E)** GSU, **(F)** CDFI, **(G)** SE, and **(H)** Mask ultrasound images in longitudinal section. GSU, grayscale ultrasound; CDFI, color Doppler flow imaging; SE, strain elastography; Mask, region of interest mask.

Four modalities of TN images were included in our research: GSU, CDFI, SE, and ROI mask (Mask) images. Each modal image was captured from both horizontal and vertical perspectives. The multimodal and double-view US images of a TN in the right lobe of a 65-year-old female patient with pathologically proven papillary carcinoma are illustrated in **Figure 2**. The Masks of the TNs were manually segmented using ImageJ (version 1.48, National Institutes of Health, USA) by two radiologists (Q.D. and Y.T.). The total data set was separated into training, validation, and test data sets, with a ratio of 4:1:1.25.

#### 2.3.2 Deep residual learning with attention block

Deep networks can extract more abstract information from low-level feature maps, which enables them to perform better than shallow networks. The residue strategy provides a skip connection to solve the degradation problem, making it possible to train a very deep network. To make full use of the multimodal image features, ResNet-50 (28) was used as the backbone for feature extraction in our method. In the ResNet-50, there is one convolutional layer and 16 residual blocks. For the essential composition of ResNet-50, a residual block is defined as follows:


(1) y=x+F(x,W)

where *x* and *y* denote the input and output feature maps of the residual block, respectively. *F* refers to the residual function, which is learned by stacked convolutional layers with different kernel sizes in the residual block. The right side of the equation is obtained by feedforward neural networks with skip connections, which allow gradients to propagate through the networks.

All available multimodal images were preprocessed to a size of 224 × 224 × 3 pixels, where 224 denotes the width and height and 3 denotes the channels of images. The training and validation data sets were randomly divided into five parts for fivefold cross-validation. Multimodal US images of the same patient were sent to the training, validation, or testing data set as one sample. During the training process, the parameters of the modal were optimized by forward and backward propagation computing until the prediction reached a high accuracy related to the ground truth. The feedforward process can be mathematically expressed as follows:


(2) $$h_{l}=R_{l}(W_{l}*h_{l-1}+b_{l})$$

where *l* denotes the number of layers. *h*_*l*_ represents the output feature map of the *l* layer with *h*_*l*−1_ as the input. *W* and *b* denote the weights and biases of the convolutional filter bank, respectively. *R* is a rectified linear activation (ReLU) function. In back propagation, the parameters of the network are updated by optimizing the following binary cross-entropy loss.

**Figure 3:**
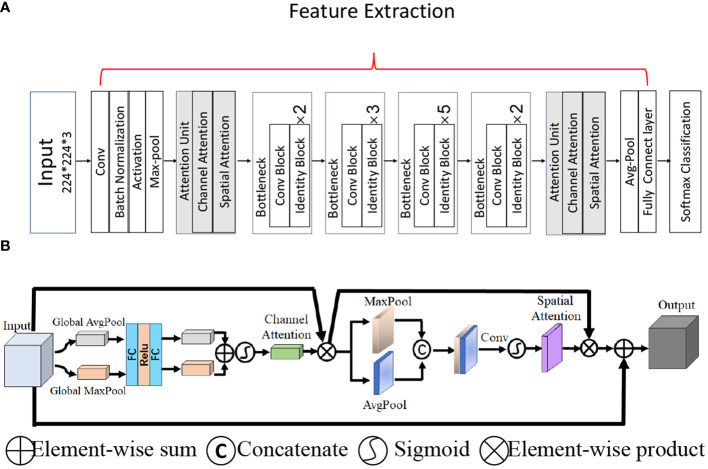
The overall network architecture. **(A)** Architecture of the backbone network ResNet-50 with two attention units. **(B)** The attention unit.

Because of the low contrast and small area of TNs in thyroid US images, it is necessary to obtain effective feature information. However, the key channels and spatial position of the lesion cannot be identified because the information obtained by the convolution operation with the kernel in ResNet is local and may fail to capture effective features from the global image. To solve this problem, we combined the convolutional bottleneck attention module (29) and ResNet-50 to learn the weights for our feature maps (**Figure 3**). Two attention units were inserted before the first and after the last residual block to obtain abstract features from both the higher and lower layers, as shown in **Figure 3A**. There are two types of attention mechanisms in the attention unit: spatial attention and channel attention, as shown in **Figure 3B**. Channel-wise attention was used to select features that could calculate the strongest channel-wise activation values. Spatial attention performs average pooling and max pooling along the channel axis on the feature map to obtain the activated feature map with a local receptive field in the spatial dimension. To complement channel attention, spatial attention was applied to find the informative region for the input feature map in the spatial dimension.

#### 2.3.3 Implementation

To establish the DL model, we used 588 benign and 550 malignant TNs with multimodal and double-view images as the data set. Furthermore, fivefold cross-validation was applied to the data sets.

**Figure 4:**
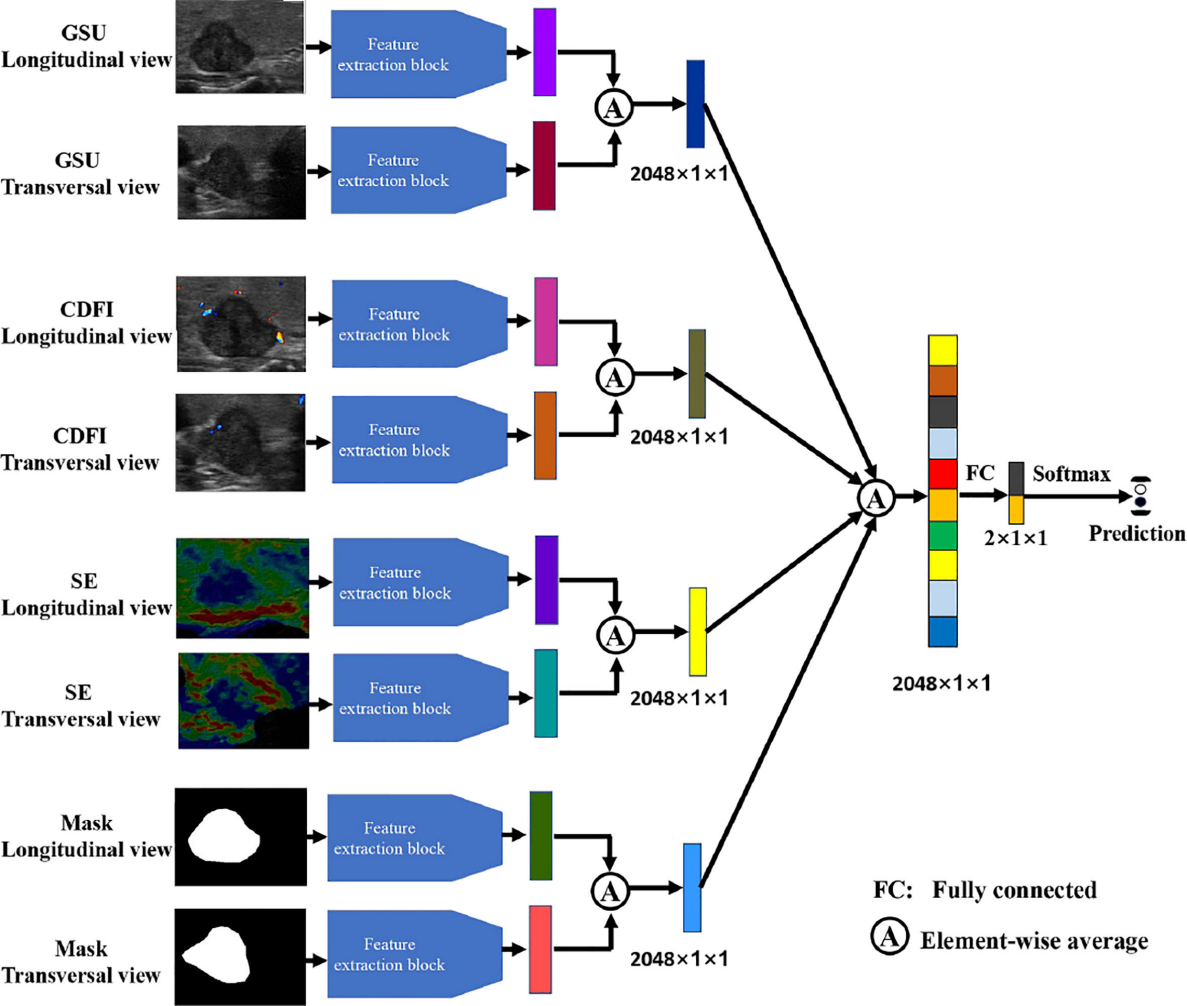
The illustration of multimodality inputs and feature fusion.

To evaluate the performance of the four types of sonography in thyroid cancer diagnosis, we performed experiments with multimodal inputs (i.e., GSU, CDFI, SE, and Mask). The four streams in **Figure 4** correspond to the four modalities. All four modalities (**Figure 4**), as well as one or multiple modalities of the same patient, were taken as the inputs. Popular ResNet-50 was used as the feature extraction backbone (**Figure 3A**). The features obtained by multiple network streams from the different modalities and views were averaged and then applied to fully connected layers to predict the classification result. In our experiments, each network stream had its own independent parameters.

The framework was implemented on a Dell-T7920 workstation equipped with an NVIDIA GeForce RTX3090 GPU and 64 GB of memory. The Adam optimization algorithm for minibatch gradient descent was used for training with a batch size of 32. The learning rate was initially set to 0.00001 and reduced by 0.1 every 30 epochs. A pretrained model was used for parameter initialization. The models with the smallest loss values within 100 training epochs were selected as the final models to generate classification results. We set the same epochs for training every modal, including the double- and single-view modes.

### 2.4 Comparing the diagnosis of the deep learning model and radiologists

In this section, we investigate the diagnostic performance of the DL models and radiologists using 228 cases from the test set. According to a survey, the diagnostic accuracy of radiologists increased when they classified the final category into either dichotomous prediction or malignant risk (9). In our study, radiologists diagnosed TNs of the test set based on multimodal US images, and the results were compared with those of the DL method. Five senior radiologists with 5–10 years of experience and five junior radiologists with 1–3 years of experience independently evaluated the TNs and were blinded the diagnosis to the postoperative pathological results. The radiologist then performed a second diagnosis based on the results of the DL and arrived at the final diagnosis. The diagnostic performance of the radiologist alone and in combination with DL assistance was compared.

### 2.5 Statistical analysis

R software (version 1.8) and MedCalc (version 11.2, Ostend, Belgium) were used to analyze the data. The data set was randomly divided into five non-overlapping groups, whereas there was no data intersection for the same subject for each group. After fivefold cross-validation, the accuracy, sensitivity, specificity, positive predictive value (PPV), negative predictive value (NPV), and area under the receiver operating characteristics curves (AUC) were obtained to evaluate the performance of the presented DL model in the test set. The Delong test results in terms of the AUC for the test data set were introduced to evaluate the statistical difference between DL based on different combined US images and radiologists with variable levels. A 95% confidence interval was used to estimate the range of these evaluation values; *p*-values of less than 0.05 (two-tailed) were considered statistically significant.

## 3 Results

### 3.1 General and ultrasonic characteristic analysis

Among the 781 patients, 135 (17.29%) were men and 646 (82.71%) were women. The mean diagnostic age of the patients was 45.92 ± 10.18 years (range, 22–67 years) for men and 48.11 ± 10.66 years (range, 21–79 years) for women. The average size of malignant TNs (12.5 ± 7.40 mm) was significantly larger than that of benign TNs (9.70 ± 6.40 mm) (*p*< 0.001) (**Table 1**).

**Table 1:** Comparing the characteristics of benign and malignant thyroid nodules.

Characteristics	All nodules	Benign	Malignant	*p*
	(n = 1138)	(n = 588)	(n = 550)	
	n (%)	n (%)	n (%)	
**Size(mm)**				< 0.001
Mean ± SD	11.2 ± 7.1	12.5 ± 7.4	9.7 ± 6.4	
(Range)	(3.0–59.0)	(3.0–44.5)	(3.0–59.0)	
**Position 1**				0.001
Left lobe	533 (46.84)	295 (50.17)	238 (43.27)	
Right lobe	585 (51.41)	290 (49.32)	295 (53.64)	
Isthmus	20 (1.76)	3 (0.51)	17 (3.09)	
**Position 2**				0.001
Upper region	250 (21.97)	117 (19.90)	133 (24.18)	
Mid region	478 (42.00)	264 (44.90)	214 (38.91)	
Lower region	390 (34.27)	204 (34.69)	186 (33.82)	
Isthmus	20 (1.76)	3 (0.51)	17 (3.09)	
**Position 3**				0.005
Shallow side	364 (31.99)	196 (33.33)	168 (30.55)	
Mid side	347 (30.49)	171 (29.08)	176 (32.00)	
Deep side	407 (35.76)	218 (37.07)	189 (34.36)	
Isthmus	20 (1.76)	3 (0.51)	17 (3.09)	
**Echotexture**				0.604
Homogeneous	113 (9.92)	61(10.40)	52(9.50)	
Heterogeneous	1025 (90.10)	527(89.60)	498(90.50)	
**Echogenicity**				< 0.001
Isoechoic or hyperechoic	359 (31.55)	307 (52.21)	52 (9.45)	
Hypoechoic	671 (58.96)	259 (44.05)	412 (74.91)	
Markedly hypoechoic	108 (9.49)	22 (3.74)	86 (15.64)	
**Composition**				< 0.001
Predominantly solid	142 (12.48)	131 (22.28)	11 (2.00)	
solid	996 (87.52)	457 (77.72)	539 (98.00)	
**Orientation**				< 0.001
Parallel	589 (51.76)	451 (76.70)	138 (25.09)	
Vertical	549 (48.24)	137 (23.30)	412 (74.91)	
**Punctate echogenic foci**				< 0.001
No punctate echogenic foci	872 (76.63)	523 (88.95)	349 (63.45)	
Punctate echogenic foci of undetermined significance	70 (6.15)	21 (3.57)	49 (8.91)	
Microcalcifications	196 (17.22)	44 (7.48)	152 (27.64)	
**Margin**				< 0.001
Circumscribed	473 (41.56)	354 (60.20)	119 (21.64)	
Ill-defined	88 (7.73)	61 (10.37)	27 (4.91)	
Irregular or extrathyroidal extension	577 (50.70)	173 (29.42)	404 (73.45)	
**Halo**				< 0.001
Absent	954 (83.83)	504 (85.71)	450 (81.82)	
Complete	53 (4.66)	38 (6.46)	15 (2.73)	
Incomplete	131 (11.51)	46 (7.82)	85 (15.45)	
**Posterior features**				< 0.001
No posterior features	932 (81.90)	530 (90.14)	402 (73.09)	
Enhancement	46 (4.04)	26 (4.42)	20 (3.64)	
Shadowing	160 (14.06)	32 (5.44)	128 (23.27)	
**Vascular distribution pattern**				0.004
Avascularity	171 (15.03)	70 (11.90)	101 (18.36)	
Peripheral vascularity	643 (56.50)	330 (56.12)	313 (56.91)	
Mainly central vascularity	121 (10.63)	71 (12.07)	50 (9.09)	
Mixed vascularity	203 (17.84)	117 (19.90)	86 (15.64)	
**Adler grade**				< 0.001
0	119 (10.46)	50 (8.50)	69 (12.55)	
1	385 (33.83)	166 (28.23)	219 (39.82)	
2–3	634 (55.71)	372 (63.27)	262 (47.64)	
**Elastography score**				< 0.001
2	559 (49.12)	386 (65.65)	173 (31.45)	
3	495 (43.50)	186 (31.63)	309 (56.18)	
4	84 (7.38)	16 (2.72)	68 (12.36)	
**C-TIRADS**				< 0.001
3	110 (9.67)	108 (18.37)	2 (0.36)	
4a	255 (22.41)	219 (37.24)	36 (6.55)	
4b	320 (28.12)	159 (27.04)	161 (29.27)	
4c	360 (31.63)	93 (15.82)	267 (48.55)	
5	93 (8.17)	9 (1.53)	84 (15.27)	

The US characteristics of the 1,138 TNs were statistically analyzed, and the results are listed in **Table 1**. Except for echotexture (*p* = 0.649), the risk features of GSU were significantly different between malignant and benign nodules (*p*< 0.05). We found some significantly different US features in the vascular distribution pattern, Adler grading, and Asteria standard in this study (*p*< 0.05).

### 3.2 Diagnostic performance of deep learning models

**Figure 5:**
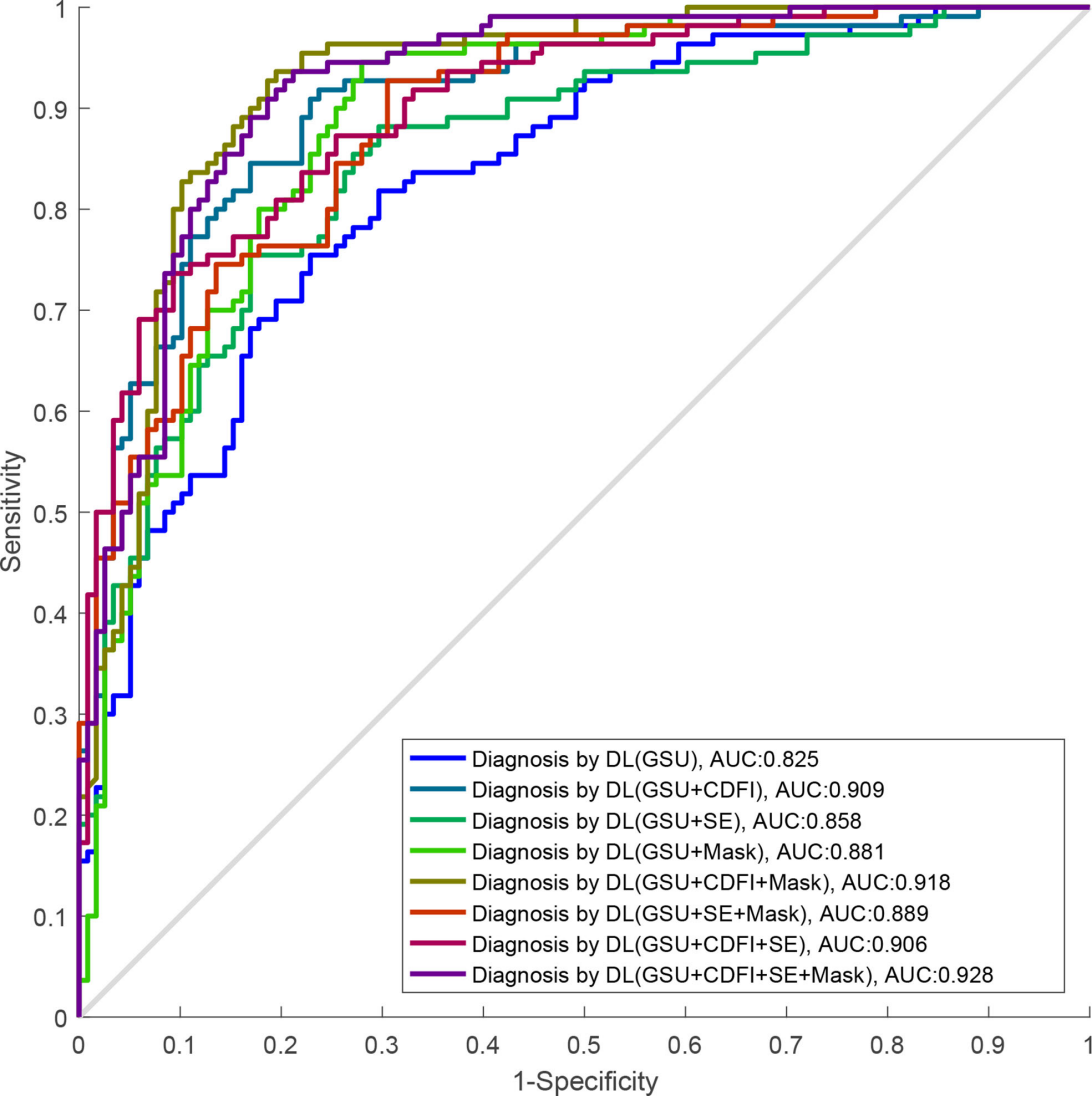
The ROC curves of DL-based single GSU and multimodality. ROC, receiver operating characteristics; DL, deep learning; GSU, gray-scale ultrasound.

The performances of the various DL models for differentiating TNs are summarized in **Table 2** and **Figure 5**. In our study, a total of eight DL models were established based on multimodal US imaging. We found that the feature fusion of images from both transverse and longitudinal sections could achieve better performance than that from a single section (**Supplementary Text S1**).

**Table 2:** Comparing the deep learning diagnostic performance based on multimodal ultrasound images.

Models	Accuracy %	Sensitivity %	Specificity %	PPV %	NPV %	AUC
G	78.9 (76.7–81.1)	77.5 (74.3–80.7)	80.3 (78.5–82.1)	78.6 (76.6–80.6)	79.3 (76.8–81.8)	0.825 (0.815–0.835)
G + C	83.8 (82.1–85.5)*	80.4 (76.5–84.3)	86.9 (86.0–87.8)**	85.2 (84.5–85.9)**	82.7 (80.0–85.4)	0.909 (0.894–0.924)**
G + E	81.8 (79.0–84.6)	75.5 (69.7–81.3)	87.6 (85.9–89.3)**	85.0 (83.2–86.8)*	79.5 (75.6–83.4)	0.858 (0.844–0.872)*
G + C + E	84.8 (82.3–87.3)*	79.8 (73.1–86.5)	89.5 (87.0–92.0)**	87.8 (85.8–89.8)**	83.0 (78.7–87.3)	0.906 (0.895–0.917)**
G + M	82.4 (81.7–83.1)*	82.9 (81.5–84.3)*	81.9 (80.8–83.0)	81.0 (80.1–81.9)	83.7 (82.6–84.8)*	0.881 (0.870–0.892)*
G + C + M	86.2 (84.4–88.0)*	86.9 (82.9–90.9)*	85.6 (84.5–86.7)*	84.9 (84.0–85.8)**	87.7 (84.5–90.9)*	0.918 (0.906–0.930)**
G + E + M	82.5 (80.5–84.5)*	82 (79.2–84.8)	82.9 (80.4–85.4)	81.7 (79.5–83.9)	83.2 (80.9–85.5)	0.889 (0.880–0.898)*
G + C + E + M	86.1 (85.5–86.7)**	84.7 (83.6–85.8)*	87.5 (86.3–88.7)**	86.3 (85.2–87.4)**	86.0 (85.2–86.8)*	0.928 (0.921–0.935)**
*p*_1_	0.264	0.208	0.501	0.889	0.220	0.001^†^
*p*_2_	0.145	0.362	0.254	0.083	0.274	0.002^†^
*p*_3_	0.504	0.894	0.099	0.045^†^	0.929	0.294
*p*_4_	0.020^†^	0.017^†^	0.196	0.061	0.014^†^	0.002^†^
*p*_5_	0.099	0.050	0.087	0.655	0.051	0.570
*p*_6_	0.685	0.083	0.013	0.051	0.143	0.215
*p*_7_	0.342	0.193	0.193	0.255	0.205	0.284

Data in parentheses have 95% confidence intervals.

US, ultrasound; G, grayscale ultrasound; C, color Doppler flow imaging; E, strain elastography; M, region of interest mask; AUC, area under the receiver operator characteristic curve; PPV, positive predictive value; NPV, negative predictive value.

p_1_ = G + C vs. G + E, p_2_ = G + E vs. G + C + E, p_3_ = G + C vs. G + C + E, p_4_ = G vs. G + M, p_5_ = G + C vs. G + C + M, p_6_ = G + E vs. G + E + M, p_7_ = G + C + E vs. G + C + E + M.

The accuracy, sensitivity, specificity, PPV, NPV, and AUC of the DL-based multimodality was statistically compared to those of the DL-based single GSU.

*p< 0.05. **p<0.001. ^†^p-Values for statistical significance (<0.05).

The AUCs of DL using multimodal US imaging (0.909 based on [G + C]US, 0.858 based on [G + E]US, and 0.906 based on [G + C + E]US) outperformed those of GSU imaging alone (0.825) (*p* = 0.014, *p*< 0.001, and *p*< 0.001, respectively). There was a statistically significant difference in the diagnosis between the (G + C)US and (G + E)US images (0.909 vs. 0.858, *p* = 0.001). However, the AUC of the DL model based on (G + C + E)US exhibited an excellent performance similar to that based on (G + C)US (0.906 vs. 0.909, *p* = 0.294), which were both markedly better than DL based on (G + E)US (0.906 vs. 0.858, *p* = 0.002; 0.909 vs. 0.858, *p* = 0.001, respectively). In addition, the accuracy, specificity, PPV, and NPV of DL based on (G + C + E)US were better than those of (G + C)US; however, only the PPV was statistically significant (87.8% vs. 85.2%, *p* = 0.045; **Supplementary Text S2**).

Furthermore, after adding the Mask feature, the diagnostic performance was obviously better than that of GSU alone (0.881 vs. 0.825, *p* = 0.002), and the AUCs of (G + C)US, (G + E)US, and (G + C + E)US were also increased (0.918 vs. 0.909, 0.889 vs. 0.858, 0.928 vs. 0.906), but without statistical differences (*p* = 0.57, *p* = 0.22, and *p* = 0.28; **Table 2**). The highest accuracy of 86.2%, sensitivity of 86.9%, and PPV of 87.8 were achieved by the DL model based on (G + C + M)US, and the highest specificity of 89.5% and NPV of 87.7% were achieved based on (G + C + E)US. The DL model using (G + C + E + M)US images achieved the best performance (AUC of 0.928), with an increase of 10.3% compared with that using a single GSU (*p*< 0.001).

### 3.3 Deep learning performance compared with radiologists

**Figure 6:**
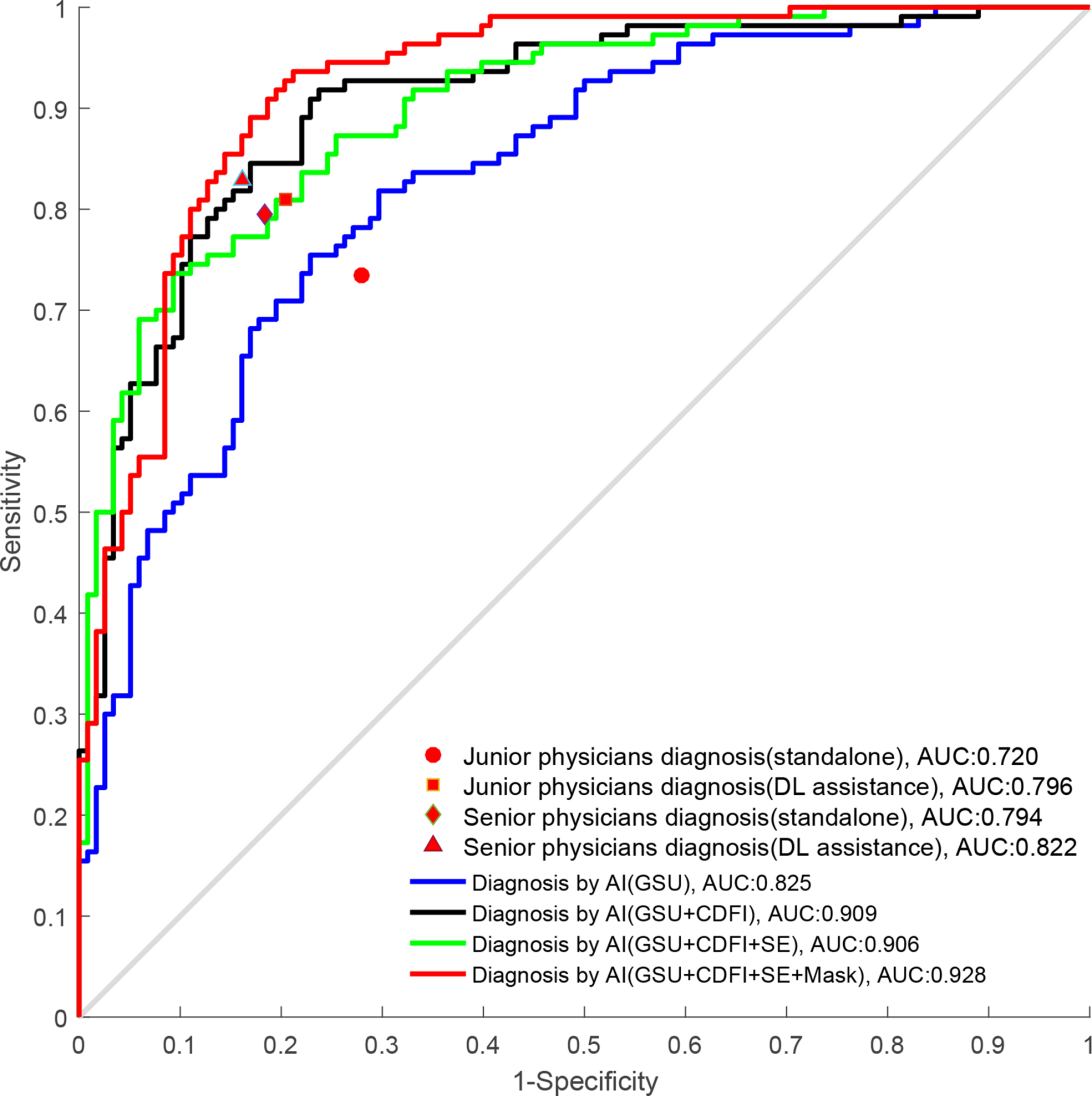
The ROC curves of DL and radiologists with different degrees of experience. ROC, receiver operating characteristics; DL, deep learning.

The diagnostic performance of radiologists with different levels of experience in differentiating malignant from benign TNs is shown in **Table 3** and **Figure 6**. When independently evaluating the TNs without DL assistance, the diagnosis of senior radiologists showed higher accuracy, specificity, and AUC than that of juniors (80.6% vs. 72.7%, *p* = 0.008; 81.7% vs. 72.0%, *p* = 0.018; 0.794 vs. 0.720, *p* = 0.002, respectively). The sensitivity (79.5%) of US diagnosis by senior radiologists was also better than that (73.5%) of junior radiologists (*p* = 0.079).

**Table 3:** The diagnostic performance of deep learning (DL), radiologists alone, and DL-assisted radiologists.

Radiologists	Accuracy %	Sensitivity %	Specificity %	PPV %	NPV %	AUC
First diagnosis without DL assistance						
Senior	80.6 (77.2–84.0)*	79.5 (75.2–83.8)	81.7 (76.4–87.0)*	80.5 (75.7–85.3)*	81.1 (77.7–84.5)*	0.794 (0.758–0.830)*
Junior	72.7 (70.8–74.6)	73.5 (70.5–76.5)	72.0 (69.8–74.2)	71.0 (69.2–72.8)	74.5 (72.2–76.8)	0.720 (0.702–0.738)
Second diagnosis with DL assistance						
Senior	83.4 (80.9–85.9)**	82.9 (79.0–86.8)**	83.9 (80.0–87.8)**	82.9 (79.5–86.3)**	84.2 (81.2–87.2)**	0.822 (0.793–0.851)**
Junior	80.2 (79.2–81.2)**	80.9 (77.9–83.9)*	79.5 (78.0–81.0)*	78.6 (77.7–79.5)**	81.8 (79.7–83.9)*	0.796 (0.786–0.806)**
*p*_1_	0.285	0.315	0.571	0.490	0.269	0.141
*p*_2_	0.070	0.489	0.099	0.066	0.290	0.465
*p*_3_	0.041†	0.081	0.177	0.083	0.054	0.512
*p*_4_	0.856	0.411	0.208	0.290	0.532	0.019†
*p*_5_	0.491	0.465	0.059	0.060	0.677	0.027†
*p*_6_	0.106	0.446	0.158	0.131	0.319	0.010†

Data in parentheses have 95% confidence intervals.

US, ultrasound; G, grayscale ultrasound; C, color Doppler flow imaging; E, strain elastography; M, region of interest mask; AUC, area under the receiver operator characteristic curve; PPV, positive predictive value; NPV, negative predictive value.

p_1_ = (senior diagnosis standalone vs. senior diagnosis with DL assistance), p_2_ = (junior diagnosis with DL assistance vs. senior diagnosis with DL assistance), p_3_ = (senior diagnosis with DL assistance vs. DL diagnosis [GSU], p_4_ = (senior diagnosis with DL assistance vs. DL diagnosis [G + C]), p_6_ = (senior diagnosis with DL assistance vs. DL diagnosis [G + C + E]), p_6_ = (senior diagnosis with DL assistance vs. DL diagnosis [G + C + E + M]).

The accuracy, sensitivity, specificity, PPV, NPV, and AUC were statistically compared with those of junior radiologists in the first diagnosis without DL assistance.

*p< 0.05. **p< 0.001. ^†^p-Values for statistical significance (<0.05).

When the DL method was added for the second diagnosis in the test set, the diagnostic performance of junior radiologists significantly increased from 0.720 to 0.796 (*p*< 0.001). The AUC of the junior radiologists in the second diagnosis was similar to that of the first diagnosis by senior radiologists (0.796 vs. 0.794) but was considered inferior (0.796 vs. 0.822), and the differences were not statistically significant (*p* > 0.05). Moreover, the DL model also had a certain auxiliary diagnostic effect and could slightly improve the diagnostic performance of senior radiologists in terms of accuracy (from 80.6% to 83.4%), which was higher than that of DL based on GSU (83.4% vs. 78.9%, *p* = 0.041). However, the AUC of senior radiologists with DL assistance was only comparable to that of DL based on a single GSU (0.822 vs. 0.825, *p* = 0.512) and significantly less than that of DL based on multimodal US images (0.822 vs. 0.858–0.928, *p*< 0.05).

## 4 Discussion

Thyroid cancer has recently become one of the most common malignancies in Chinese women (2). US was the first choice for the examination of thyroid lesions, and TNs were diagnosed on US imaging by radiologists according to TI-RADS. Each guideline has its strengths and weaknesses; for example, the American Thyroid Association 2015 guideline showed better diagnostic efficiency in evaluating TNs >1 cm, the TIRADS issued by the American College of Radiology in 2017 had more advantages in reducing unnecessary biopsy operations, and TNs were well diagnosed by radiologists according to C-TIRADS, achieving a higher performance (6–8). However, the diagnostic results were susceptible to operator dependency, probe, and US equipment variability. FNA is a comparatively accurate method for differentiating TNs preoperatively, but it was reported that approximately 20% of FNA samples obtained had ambiguous results (15–17). AI not only solves the complex problem of the US risk stratification system but also reduces intra- and interobserver variability in US diagnosis (23, 30).

Nevertheless, most applications of DL in the diagnosis of TNs have been conducted based on single GSU imaging or single-view sections, limiting access to image information to a certain extent (21, 31–33). In addition to GSU, radiologists also referred to CDFI and elastography for obtaining the blood flow and hardness information of TNs to assist the GSU diagnosis clinically and make a diagnosis after a comprehensive analysis. In terms of the statistical analysis in our study, a higher elastic score was markedly correlated with malignant TNs, confirming that malignant TNs tend to be hard. The differences in vascular distribution pattern and Adler grade were statistically significant in TNs, and malignant TNs tended to be less or lacked blood flow. In addition, many studies have verified the effectiveness of combined or multimodal US imaging in the differentiation of TNs visually (11, 13, 24, 25). Therefore, based on multimodal US images of TNs obtained from transverse and longitudinal sections, new DL models were used to distinguish benign from malignant TNs in our study.

In our study, the diagnostic performance of GSU (0.825) was comparable to that of previous studies (AUC of 0.788 and 0.829) (32, 33). However, the DL models using combined or multimodal US images achieved a better performance (0.858–0.928) than those using GSU alone (0.825) (*p*< 0.05). Notably, the AUC of the DL model based on GSU alone was also greatly improved after adding CDFI (0.825 vs. 0.909, *p*< 0.001). In a related study, Baig et al. quantified the regional blood flow indices of TNs, and the diagnostic accuracy of GSU features was increased from 58.6% to 79.3% when combined with CDFI (*p*< 0.05) (34). The DL models in our study provided consistent and repeatable results and outperformed conventional machine learning-based methods with a specificity of 86.9%, a PPV of 85.2%, and an accuracy of 83.8%. As for the improvement in diagnostic efficiency after adding CDFI, we found that it may be due to the attention mechanism algorithm applied in this study, which could obtain richer and more objective features that were previously unrecognized visually by learning the information of CDFI images autonomously. We also demonstrated that SE imaging helped improve the diagnosis of DL based on GSU (0.825 vs. 0.858, *p*< 0.05). However, the AUC of the DL model based on (G + E)US was markedly less than that based on (G + C)US (0.858 vs. 0.909, *p* = 0.001). Additionally, there were no significant differences between (G + C + E)US and (G + C)US (0.906 vs. 0.909, *p* = 0.294). Therefore, our study confirmed that CDFI played a more substantial role in distinguishing TNs than SE in our study, and the less obvious advantages of SE may be associated with the subjectivity of the collecting process of SE images.

Adding a Mask containing the contour information of the TNs was found to help improve the diagnostic performance of DL models based on GSU (from 0.825 to 0.881), (G + C)US (from 0.909 to 0.918), (G + E)US (from 0.858 to 0.889), and (G + C + E)US (from 0.906 to 0.928), indicating that effective delineation of the nodular boundaries in US images played an important role in characterizing TNs. The best AUC of 0.928 was achieved by DL using (G + C + E + M)US. The highest specificity (89.5%) and PPV (87.8%) were achieved by DL based on (G + C + E)US, which could play a primary role in avoiding overdiagnosis and helping reduce unnecessary biopsies for the diagnosis of TNs, whereas the highest sensitivity (86.9%) and NPV (87.7%) of great clinical significance for screening out malignant TNs and avoiding misdiagnosis were achieved by DL based on (G + C + M)US. In summary, the performance of DL models based on multimodal US imaging was superior to that based on a single GSU, which supports our assumption that multimodal US could provide more comprehensive and effective information for TN diagnosis.

In clinical practice, US diagnosis by radiologists cannot be completely replaced by AI technology, and a final diagnosis should be made by radiologists. Therefore, we compared the performance of the DL method for differentiating TNs with that of visual diagnosis by radiologists and further explored the auxiliary role of DL for radiologists’ diagnosis. Compared with the first diagnosis of TNs visually by junior radiologists, there was a significant improvement in the second diagnosis with DL assistance (0.720 vs. 0.796, *p*< 0.001), which could be comparable to that of the seniors in the first diagnosis (0.796 vs. 0.794, *p* > 0.05). Moreover, the DL method could also provide an auxiliary diagnosis for senior radiologists in terms of accuracy (from 80.6% to 83.4%), which was superior to DL based on GSU alone (83.4% vs. 78.9%, *p* = 0.041). It has been proven that DL can assist clinical radiologists in improving diagnostic ability and increasing confidence, especially for juniors with less experience. In a study by Peng et al. (35), the DL-assisted method also improved the AUC of radiologists in diagnosing TNs from 0.837 to 0.875 (*p*< 0.001).

Nevertheless, there were no significant diagnostic differences with and without DL assistance for senior radiologists (0.794 vs. 0.822, *p* = 0.141). It seems that DL-aided diagnosis was less effective for senior than junior radiologists, which may be related to the fact that senior radiologists were more likely to rely on their own clinical experience. Our analysis may also be due to the fact that, by using DL models, we sacrificed interpretability for robust and complex imaging features with greater generalizability. Furthermore, DL technology obtained results based on features that were learned and extracted independently rather than on predefined handcrafted features, where the process was abstract and incomprehensible, leading to distrust by the radiologists. To resolve the visualization of DL learning and decision processes, Kim et al. (36) applied Grad-CAM to generate output images overlaid with heat maps to achieve visual interpretability. Meanwhile, Zhou et al. (20) found that the adjacent parenchyma of TNs is critical for classification by visual interpretability of DL.

By comparing the radiologists’ and DL’s diagnostic efficacy, we found that senior radiologists with DL assistance only had a diagnosis comparable to the DL model based on GSU in terms of AUC (0.822 vs. 0.825, *p* = 0.512), which could not be compared with the diagnosis based on multimodal US imaging (0.822 vs. 0.858–0.928, *p*< 0.05), effectively demonstrating the excellent clinical value of the DL method, especially for multimodal US imaging, with potential for further development and application. Radiologists may be affected by fatigue and other factors in daily work, whereas AI can run on its own and has the characteristics of indefatigability with stable and high diagnostic efficiency.

Our study has several advantages. To our knowledge, this is the first study in which the attention mechanism-guided residual network was used to construct a variety of DL models based on different US imaging combinations. The objects in our study were TNs of the C-TIRADS 3–5 categories, which are more suitable for clinical diagnosis difficulties and extend the scope of clinical application. We have verified that DL models based on multimodal US can assist radiologists in improving diagnostic performance, especially for those with less experience, and postoperative pathological results were used as the gold standard for statistical analysis in this study, which was more objective than the studies using cytological pathological results (20, 31). Our case set included relatively more samples and achieved a balance between benign and malignant TNs, which could effectively reduce the diagnostic bias compared with a previous study (18).

This study had some limitations. First, the main limitation of our study was that the data were retrospectively derived from a single center, and additional external validation or multicenter studies are needed to refine our study. Second, the images in this study were static images stored in a compressed format, which may have led to some potential image features not being mined. Therefore, dynamic images or raw radiofrequency signals should be included in future studies. Third, the visualization of the DL proposed in this study was not achieved. Visualization of the DL process could be conducted to make the results more reliable in subsequent studies. More technologies could be included, such as shear wave elastography, superb microvascular imaging, and contrast-enhanced US.

In conclusion, the DL model based on multimodal US images can achieve a high diagnostic value in the differential diagnosis of benign and malignant TNs of C-TIRADS 3–5 categories, aid second-opinion provision, and improve the diagnostic ability for radiologists, which is of great significance for clinical decision-making.

## Data availability statement

The original contributions presented in the study are included in the article/**Supplementary Material**. Further inquiries can be directed to the corresponding authors.

## Ethics statement

The studies involving human participants were reviewed and approved by the Ethics Committee of the Second Affiliated Hospital of Harbin Medical University. Written informed consent for participation was not required for this study in accordance with the national legislation and the institutional requirements.

## Author contributions

YY and JT conceived and designed the study. YT, QD, and HK collected the clinical and image data and performed image preprocessing. YT, LZ, and WY analyzed and evaluated the ultrasonographic features of thyroid nodules. YY and WQ provided a deep learning algorithm and built the models. YY analyzed the image data and performed statistical analysis. YT, YY, and TW wrote the manuscript. JT, XX, and XL reviewed and edited the manuscript. All authors have contributed to the manuscript and approved the submitted version.

## Funding

This work was supported by the National Key R&D Program of China (2018YFA0701400), the Shenzhen Universities Stabilization Support Program (20200812144239001), the National Nature Science Foundation Grants of China (12174267, 62022086, 11874382, and 81827802), the Shenzhen Foundation (JCYJ20200109114237902, SGDX2020110309400200, and ZDSYS201802061806314), CAS research projects (KFJ-PTXM-012 and 2011DP173015), the Natural Science Foundation of Guangdong Province (2022A1515011343, 2020B1111130002, and 2020B1212060051), and the Youth Innovation Promotion Association (CAS 2018391).

## Acknowledgments

We would like to thank the ultrasound doctors in the Department of Ultrasound, The Second Affiliated Hospital of Harbin Medical University, Harbin, for participating in the diagnosis of thyroid nodules.

## Conflict of interest

The authors declare that the research was conducted in the absence of any commercial or financial relationships that could be construed as a potential conflict of interest.

## Publisher’s note

All claims expressed in this article are solely those of the authors and do not necessarily represent those of their affiliated organizations, or those of the publisher, the editors and the reviewers. Any product that may be evaluated in this article, or claim that may be made by its manufacturer, is not guaranteed or endorsed by the publisher.

## References

[1] Haugen BR Alexander EK Bible KC Doherty GM Mandel SJ Nikiforov YE. American Thyroid association management guidelines for adult patients with thyroid nodules and differentiated thyroid cancer the American thyroid association guidelines task force on thyroid nodules and differentiated thyroid cancer. Thyroid (2016) 26:1–133. doi: 10.1089/thy.2015.0020 26462967 PMC4739132

[2] Chen W Zheng R Baade PD Zhang S Zeng H Bray F. Cancer statistics in China, 2015. CA Cancer J Clin (2016) 66:115–32. doi: 10.3322/caac.21338 26808342

[3] Pacini F Castagna MG Brilli L Pentheroudakis G Grp EGW. Thyroid cancer: ESMO clinical practice guidelines for diagnosis, treatment and follow-up. Ann Oncol (2012) 23:110–9. doi: 10.1093/annonc/mds230 22997443

[4] Kwak JY Han KH Yoon JH Moon HJ Son EJ Park SH. Thyroid imaging reporting and data system for US features of nodules: A step in establishing better stratification of cancer risk. Radiology (2011) 260:892–9. doi: 10.1148/radiol.11110206 21771959

[5] Tessler FN Middleton WD Grant EG Hoang JK Berland LL Teefey SA. ACR thyroid imaging, reporting and data system (TI-RADS): White paper of the ACR TI-RADS committee. J Am Coll Radiol (2017) 14:587–95. doi: 10.1016/j.jacr.2017.01.046 28372962

[6] Zhou J Song Y Zhan W Wei X Zhang S Zhang R. Thyroid imaging reporting and data system (TIRADS) for ultrasound features of nodules: Multicentric retrospective study in China. Endocrine (2021) 72:157–70. doi: 10.1007/s12020-020-02442-x 32852733

[7] Liu H Ma AL Zhou YS Yang DH Ruan JL Liu XD. Variability in the interpretation of grey-scale ultrasound features in assessing thyroid nodules: A systematic review and meta-analysis. Eur J Radiol (2020) 129:109050. doi: 10.1016/j.ejrad.2020.109050 32447147

[8] Choi SH Kim EK Kwak JY Kim MJ Son EJ. Interobserver and intraobserver variations in ultrasound assessment of thyroid nodules. Thyroid (2010) 20:167–72. doi: 10.1089/thy.2008.0354 19725777

[9] Park CS Kim SH Jung SL Kang BJ Kim JY Choi JJ. Observer variability in the sonographic evaluation of thyroid nodules. J Clin Ultrasound (2010) 38:287–93. doi: 10.1002/jcu.20689 20544863

[10] Lee HJ Yoon DY Seo YL Kim JH Baek S Lim KJ. Intraobserver and interobserver variability in ultrasound measurements of thyroid nodules. J Ultrasound Med (2018) 37:173–8. doi: 10.1002/jum.14316 28736947

[11] Chaigneau E Russ G Royer B Bigorgne C Bienvenu-Perrard M Rouxel A. TIRADS score is of limited clinical value for risk stratification of indeterminate cytological results. Eur J Endocrinol (2018) 179:13–20. doi: 10.1530/EJE-18-0078 29703794

[12] Seo H Na DG Kim JH Kim KW Yoon JW. Ultrasound-based risk stratification for malignancy in thyroid nodules: A four-tier categorization system. Eur Radiol (2015) 25:2153–62. doi: 10.1007/s00330-015-3621-7 25680723

[13] Pei SF Cong SZ Zhang B Liang CH Zhang L Liu JJ. Diagnostic value of multimodal ultrasound imaging in differentiating benign and malignant TI-RADS category 4 nodules. Int J Clin Oncol (2019) 24:632–9. doi: 10.1007/s10147-019-01397-y PMC6525125 30825007

[14] Ha EJ Na DG Baek JH Sung JY Kim JH Kang SY. US Fine-needle aspiration biopsy for thyroid malignancy: Diagnostic performance of seven society guidelines applied to 2000 thyroid nodules. Radiology (2018) 287:893–900. doi: 10.1148/radiol.2018171074 29465333

[15] Singh Ospina N Brito JP Maraka S Espinosa de Ycaza AE Rodriguez-Gutierrez R Gionfriddo MR. Diagnostic accuracy of ultrasound-guided fine needle aspiration biopsy for thyroid malignancy: Systematic review and meta-analysis. Endocrine (2016) 53:651–61. doi: 10.1007/s12020-016-0921-x 27071659

[16] Theoharis CGA Schofield KM Hammers L Udelsman R Chhieng DC. The Bethesda thyroid fine-needle aspiration classification system: Year 1 at an academic institution. Thyroid (2009) 19:1215–23. doi: 10.1089/thy.2009.0155 19888859

[17] Mathur A Weng J Moses W Steinberg SM Rahbari R Kitano M. A prospective study evaluating the accuracy of using combined clinical factors and candidate diagnostic markers to refine the accuracy of thyroid fine needle aspiration biopsy. Surgery (2010) 148:1170–6. doi: 10.1016/j.surg.2010.09.025 PMC3052943 21134548

[18] Liu Z Zhong SB Liu Q Xie CX Dai YZ Peng C. Thyroid nodule recognition using a joint convolutional neural network with information fusion of ultrasound images and radiofrequency data. Eur Radiol (2021) 31:5001–11. doi: 10.1007/s00330-020-07585-z 33409774

[19] Wang K Lu X Zhou H Gao Y Zheng J Tong M. Deep learning radiomics of shear wave elastography significantly improved diagnostic performance for assessing liver fibrosis in chronic hepatitis b: A prospective multicentre study. Gut (2019) 68:729–41. doi: 10.1136/gutjnl-2018-316204 PMC6580779 29730602

[20] Zhou H Jin YH Dai L Zhang MW Qiu YQ Wang K. Differential diagnosis of benign and malignant thyroid nodules using deep learning radiomics of thyroid ultrasound images. Eur J Radiol (2020) 127:108992. doi: 10.1016/j.ejrad.2020.108992 32339983

[21] Kwon SW Choi IJ Kang JY Jang WI Lee GH Lee MC. Ultrasonographic thyroid nodule classification using a deep convolutional neural network with surgical pathology. J Digit Imaging (2020) 33:1202–8. doi: 10.1007/s10278-020-00362-w PMC7572950 32705433

[22] Akkus Z Cai J Boonrod A Zeinoddini A Weston AD Philbrick KA. A survey of deep-learning applications in ultrasound: Artificial intelligence-powered ultrasound for improving clinical workflow. J Am Coll Radiol (2019) 16:1318–28. doi: 10.1016/j.jacr.2019.06.004 31492410

[23] Sorrenti S Dolcetti V Radzina M Bellini MI Frezza F Munir K. Artificial intelligence for thyroid nodule characterization: Where are we standing? Cancers (2022) 14(14). doi: 10.3390/cancers14143357 PMC9315681 35884418

[24] Colakoglu B Yildirim D Alis D Ucar G Samanci C Ustabasioglu FE. Elastography in distinguishing benign from malignant thyroid nodules. J Clin Imag Sci (2016) 6:51. doi: 10.4103/2156-7514.197074 PMC5209857 28123841

[25] Sorrenti S Dolcetti V Fresilli D Del Gaudio G Pacini P Huang PT. The role of CEUS in the evaluation of thyroid cancer: From diagnosis to local staging. J Clin Med (2021) 10(19). doi: 10.3390/jcm10194559 PMC8509399 34640574

[26] Adler DD Carson PL Rubin JM Quinn-Reid D. Doppler Ultrasound color flow imaging in the study of breast cancer: Preliminary findings. Ultrasound Med Biol (1990) 16:553–9. doi: 10.1016/0301-5629(90)90020-d 2238263

[27] Asteria C Giovanardi A Pizzocaro A Cozzaglio L Morabito A Somalvico F. US-Elastography in the differential diagnosis of benign and malignant thyroid nodules. Thyroid (2008) 18:523–31. doi: 10.1089/thy.2007.0323 18466077

[28] He KM Zhang XY Ren SQ Sun J Ieee. (2016). Deep residual learning for image recognition, in: 2016 IEEE Conference on Computer Vision and Pattern Recognition (CVPR) (Seattle, WA) 27-30. doi: 10.1109/cvpr.2016.90

[29] Woo SH Park J Lee JY Kweon IS eds. (2018). CBAM: convolutional block attention module, in: Lecture Notes in Computer Science. (Munich, Germany: 15th Eur Conference on Computer Vision (ECCV)) (2018):3–19. doi: 10.1007/978-3-030-01234-2_1

[30] Ha EJ Baek JH Na DG. Risk stratification of thyroid nodules on ultrasonography: Current status and perspectives. Thyroid (2017) 27:1463–8. doi: 10.1089/thy.2016.0654 28946821

[31] Li XC Zhang S Zhang Q Wei X Pan Y Zhao J. Diagnosis of thyroid cancer using deep convolutional neural network models applied to sonographic images: A retrospective, multicohort, diagnostic study. Lancet Oncol (2019) 20:193–201. doi: 10.1016/S1470-2045(18)30762-9 30583848 PMC7083202

[32] Wu GG Lv WZ Yin R Xu JW Yan YJ Chen RX. Deep learning based on ACR TI-RADS can improve the differential diagnosis of thyroid nodules. Front Oncol (2021) 11:575166. doi: 10.3389/fonc.2021.575166 33987082 PMC8111071

[33] Zhang YC Wu Q Chen YT Wang Y. A clinical assessment of an ultrasound computer-aided diagnosis system in differentiating thyroid nodules with radiologists of different diagnostic experience. Front Oncol (2020) 10:557169. doi: 10.3389/fonc.2020.557169 33042840 PMC7518212

[34] Baig FN van Lunenburg JTJV Liu SYW Yip SP Law HKW Ying M. Computer-aided assessment of regional vascularity of thyroid nodules for prediction of malignancy. Sci Rep (2017) 7:14350. doi: 10.1038/s41598-017-14432-7 29084994 PMC5662577

[35] Peng S Liu YH Lv WM Liu LZ Zhou Q Yang H. Deep learning-based artificial intelligence model to assist thyroid nodule diagnosis and management: A multicentre diagnostic study. Lancet Digit Health (2021) 3:e250–9. doi: 10.1016/S2589-7500(21)00041-8 33766289

[36] Kim YJ Choi Y Hur SJ Park KS Kim HJ Seo M. Deep convolutional neural network for classification of thyroid nodules on ultrasound: Comparison of the diagnostic performance with that of radiologists. Eur J Radiol (2022) 152:110335. doi: 10.1016/j.ejrad.2022.110335 35512512

