# Supplementary material for: Deep learning for the diagnosis of suspicious thyroid nodules based on multimodal ultrasound images
Source: Front Oncol. 2022 Nov 8;12:1012724. doi: 10.3389/fonc.2022.1012724 (PMC9680169; doi:10.3389/fonc.2022.1012724)
Supplement: Supplementary file 1 [file DataSheet_1.docx]

Supplementary Material

# Supplementary Data

# The US characteristics of 1138 included TNs were statistically analyzed in this study, The features of extremely hypoechoic, solid composition, irregular/extra-thyroid invasion margin and microcalcification were associated with malignant TNs , and different guidelines assigned to these five categories. The echotexture of most TNs tended to be heterogeneous, being of no effect for the differentiation of benign and malignant TNs . At present, different studies have different evaluation standards for the ultrasound feature of halo, especially for that the specific threshold of distinguishing thin and thick halo was 1mm or 2 mm still was not unified. In this study, the uniformity of the halo thickness was used to evaluate TNs, and it was found that the halo thickness of malignant TNs was usually uneven. In this study, all ultrasonic image features of TNs in the guidelines were screened and analyzed one by one to ensure a more comprehensive and detailed evaluation. As previous studies had shown that there was no evidence of an obvious relationship between the number of TNs and the degree of malignancy, the analysis of the number of TNs was not included.

# Supplementary Figures and Tables

# Supplementary table 1 |Comparison of DL diagnostic performance based on ultrasound images in single or double view

| Model | Section | loss | Accuracy | Sensitivity | Specificity | PPV | NPV | AUC |
| --- | --- | --- | --- | --- | --- | --- | --- | --- |
| GSU | transverse section | 0.551 | 0.73 | 0.745 | 0.715 | 0.711 | 0.754 | 0.802 |
|  | longitudinal section | 0.622 | 0.717 | 0.662 | 0.768 | 0.727 | 0.71 | 0.74 |
|  | double section | 0.739 | 0.789 | 0.775 | 0.803 | 0.786 | 0.793 | 0.825 |
| GSU+CDFI | transverse section | 0.573 | 0.808 | 0.816 | 0.8 | 0.793 | 0.824 | 0.886 |
|  | longitudinal section | 0.662 | 0.782 | 0.796 | 0.769 | 0.763 | 0.802 | 0.838 |
|  | double section | 0.656 | 0.838 | 0.804 | 0.869 | 0.852 | 0.827 | 0.909 |
| GSU+SE | transverse section | 0.626 | 0.782 | 0.785 | 0.778 | 0.768 | 0.797 | 0.829 |
|  | longitudinal section | 0.702 | 0.746 | 0.689 | 0.8 | 0.763 | 0.735 | 0.789 |
|  | double section | 0.65 | 0.818 | 0.755 | 0.876 | 0.85 | 0.795 | 0.858 |
| GSU+Mask | transverse section | 0.697 | 0.775 | 0.793 | 0.758 | 0.754 | 0.801 | 0.853 |
|  | longitudinal section | 0.74 | 0.766 | 0.755 | 0.776 | 0.759 | 0.773 | 0.826 |
|  | double section | 0.775 | 0.824 | 0.829 | 0.819 | 0.81 | 0.837 | 0.881 |
| Total Fusion with double sections | GSU+CDFI+SE | 0.663 | 0.848 | 0.798 | 0.895 | 0.878 | 0.83 | 0.906 |
|  | GSU+CDFI+Mask | 0.707 | 0.862 | 0.869 | 0.856 | 0.849 | 0.877 | 0.918 |
|  | GSU+SE+Mask | 0.73 | 0.825 | 0.82 | 0.829 | 0.817 | 0.832 | 0.889 |
|  | GSU+CDFI+SE+Mask | 0.711 | 0.861 | 0.847 | 0.875 | 0.863 | 0.86 | 0.928 |

# Supplementary table 2 |Comparison of DL diagnostic performance based on multimodal ultrasound images

| Model | Accuracy% | Sensitivity% | Specificity% | PPV% | NPV% | AUC |
| --- | --- | --- | --- | --- | --- | --- |
| G | 78.9 (76.7,81.1) | 77.5 (74.3,80.7) | 80.3 (78.5,82.1) | 78.6 (76.6,80.6) | 79.3 (76.8,81.8) | 0.825 (0.815,0.835) |
| G+C | 83.8 (82.1,85.5)* | 80.4 (76.5,84.3) | 86.9 (86.0,87.8)** | 85.2 (84.5,85.9)** | 82.7 (80.0,85.4) | 0.909 (0.894,0.924)** |
| G+E | 81.8 (79.0,84.6) | 75.5 (69.7,81.3) | 87.6 (85.9,89.3)** | 85.0 (83.2,86.8)* | 79.5 (75.6,83.4) | 0.858 (0.844,0.872)* |
| G+C+E | 84.8 (82.3,87.3)* | 79.8 (73.1,86.5) | 89.5 (87.0,92.0)** | 87.8 (85.8,89.8)** | 83.0 (78.7,87.3) | 0.906 (0.895,0.917)** |
| G+M | 82.4 (81.7,83.1)* | 82.9 (81.5,84.3)* | 81.9 (80.8,83.0) | 81.0 (80.1,81.9) | 83.7 (82.6,84.8)* | 0.881 (0.870,0.892)* |
| G+C+M | 86.2 (84.4,88.0)* | 86.9 (82.9,90.9)* | 85.6 (84.5,86.7)* | 84.9 (84.0,85.8)** | 87.7 (84.5,90.9)* | 0.918 (0.906,0.930)** |
| G+E+M | 82.5 (80.5,84.5)* | 82 (79.2,84.8) | 82.9 (80.4,85.4) | 81.7 (79.5,83.9) | 83.2 (80.9,85.5) | 0.889 (0.880,0.898)* |
| G+C+E+M | 86.1 (85.5,86.7)** | 84.7 (83.6,85.8)* | 87.5 (86.3,88.7)** | 86.3 (85.2,87.4)** | 86.0 (85.2,86.8)* | 0.928 (0.921,0.935)** |
| P-value(G vs. G+C) | 0.0096 | 0.2962 | <0.001 | <0.001 | 0.1131 | <0.001 |
| P-value(G vs. G+E) | 0.1658 | 0.5727 | <0.001 | 0.0014 | 0.9412 | 0.014 |
| P-value(G vs. G+M) | 0.0203 | 0.0173 | 0.1955 | 0.0609 | 0.0143 | 0.002 |
| P-value(G vs. G+C+E) | 0.0085 | 0.5503 | <0.001 | <0.001 | 0.1904 | <0.001 |
| P-value(G vs. G+C+M) | 0.006 | 0.007 | 0.0011 | <0.001 | 0.0041 | <0.001 |
| P-value(G vs. G+E+M) | 0.0482 | 0.0735 | 0.1396 | 0.0677 | 0.0559 | 0.001 |
| P-value(G vs. G+C+E+M) | <0.001 | 0.0032 | <0.001 | <0.001 | 0.0012 | <0.001 |
| P-value(G+C vs. G+E) | 0.2636 | 0.2076 | 0.5009 | 0.8893 | 0.2195 | 0.001 |
| P-value(G+C vs. G+M) | 0.1848 | 0.2663 | <0.001 | <0.001 | 0.517 | 0.008 |
| P-value(G+C vs. G+C+E) | 0.5041 | 0.8944 | 0.0988 | 0.0454 | 0.9285 | 0.294 |
| P-value(G+C vs. G+C+M) | 0.0988 | 0.0505 | 0.0874 | 0.6553 | 0.0509 | 0.57 |
| P-value(G+C vs. G+E+M) | 0.3659 | 0.5245 | 0.0146 | 0.0186 | 0.79 | 0.036 |
| P-value(G+C vs. G+C+E+M) | 0.0309 | 0.068 | 0.5334 | 0.1127 | 0.0523 | 0.981 |
| P-value(G+E vs. G+M) | 0.6782 | 0.041 | p<0.001 | 0.0043 | 0.0724 | 0.563 |
| P-value(G+E vs. G+C+E) | 0.1452 | 0.3623 | 0.2537 | 0.0825 | 0.2735 | 0.002 |
| P-value(G+E vs. G+C+M) | 0.0402 | 0.013 | 0.0781 | 0.9081 | 0.013 | 0.004 |
| P-value(G+E vs. G+E+M) | 0.6848 | 0.083 | 0.0133 | 0.0512 | 0.1426 | 0.215 |
| P-value(G+E vs. G+C+E+M) | 0.0167 | 0.0154 | 0.8824 | 0.2551 | 0.0118 | <0.001 |
| P-value(G+M vs. G+C+E) | 0.1017 | 0.4019 | <0.001 | <0.001 | 0.7383 | 0.059 |
| P-value(G+M vs. G+C+M) | 0.0324 | 0.1012 | 0.0015 | <0.001 | 0.0534 | 0.002 |
| P-value(G+M vs. G+E+M) | 0.9286 | 0.5951 | 0.484 | 0.5549 | 0.6969 | 0.441 |
| P-value(G+M vs. G+C+E+M) | <0.001 | 0.0828 | <0.001 | <0.001 | 0.01 | <0.001 |
| P-value(G+C+E vs. G+C+M) | 0.223 | 0.1112 | 0.0243 | 0.0349 | 0.1235 | 0.721 |
| P-value(G+C+E vs. G+E+M) | 0.1852 | 0.5716 | 0.0062 | 0.0041 | 0.9189 | 0.13 |
| P-value(G+C+E vs. G+C+E+M) | 0.3422 | 0.1926 | 0.193 | 0.2551 | 0.2051 | 0.284 |
| P-value(G+C+M vs. G+E+M) | 0.0469 | 0.085 | 0.0784 | 0.0289 | 0.058 | 0.021 |
| P-value(G+C+M vs. G+C+E+M) | 0.3832 | 0.3283 | 0.0583 | 0.0769 | 0.3585 | 0.484 |
| P-value(G+E+M vs. G+C+E+M) | 0.0081 | 0.1172 | 0.0112 | 0.006 | 0.0512 | 0.001 |

Statistical quantifications were demonstrated with 95% confidence interval.

Abbreviations: US, ultrasound; G, gray-scale ultrasound; C, color Doppler flow imaging; E, strain elastography; M, region of interest mask; AUC, area under the receiver operator characteristic curve; PPV, positive predictive value; NPV, negative predictive value.

The accuracy, sensitivity, specificity, PPV, NPV and AUC of DL based multimodality were statistically compared to those of DL based single GSU, respectively (*, P<0.05; **, P<0.001). ^†^P values for statistical significance(<0.05).

**Supplementary table 3 |The diagnostic performance of DL, radiologists alone, and DL-assisted radiologists.**

| Radiologists | Accuracy% | Sensitivity% | Specificity% | PPV% | NPV% | AUC |
| --- | --- | --- | --- | --- | --- | --- |
| First diagnosis without DL-assistance | | | | | | |
| Senior | 80.6 (77.2,84.0)* | 79.5 (75.2,83.8) | 81.7 (76.4,87.0)* | 80.5 (75.7,85.3)* | 81.1 (77.7,84.5)* | 0.794 (0.758,0.83)* |
| Junior | 72.7 (70.8,74.6) | 73.5 (70.5,76.5) | 72.0 (69.8,74.2) | 71.0 (69.2,72.8) | 74.5 (72.2,76.8) | 0.72 (0.702,0.738) |
| Second diagnosis with DL-assistance | | | | | | |
| Senior | 83.4 (80.9,85.9)** | 82.9 (79.0,86.8)** | 83.9 (80.0,87.8)** | 82.9 (79.5,86.3)** | 84.2 (81.2,87.2)** | 0.822 (0.793,0.851)** |
| Junior | 80.2 (79.2,81.2)** | 80.9 (77.9,83.9)* | 79.5 (78.0,81.0)* | 78.6 (77.7,79.5)** | 81.8 (79.7,83.9)* | 0.796 (0.786,0.806)** |
| P-value(Junior diagnosis standalone vs. AI diagnosis(G)) | 0.0043 | 0.1303 | <0.001 | <0.001 | 0.0313 | 0.023 |
| P-value(Junior diagnosis standalone vs. AI diagnosis(G+C)) | <0.001 | 0.0299 | <0.001 | <0.001 | 0.0027 | <0.001 |
| P-value(Junior diagnosis standalone vs. AI diagnosis(G+C+E)) | <0.001 | 0.1322 | <0.001 | <0.001 | 0.0105 | <0.001 |
| P-value(Junior diagnosis standalone vs. AI diagnosis(G+C+E+M)) | <0.001 | <0.001 | <0.001 | <0.001 | <0.001 | <0.001 |
| P-value(Junior diagnosis standalone vs. Junior diagnosis with AI assistant) | <0.001 | 0.0146 | 0.0013 | <0.001 | 0.0034 | <0.001 |
| P-value(Junior diagnosis standalone vs. Senior diagnosis standalone) | 0.008 | 0.0791 | 0.0183 | 0.0119 | 0.0221 | 0.002 |
| P-value(Junior diagnosis standalone vs. Senior diagnosis with AI assistant) | <0.001 | 0.009 | 0.0016 | <0.001 | 0.002 | <0.001 |
| P-value(Senior diagnosis standalone vs. AI diagnosis(G)) | 0.478 | 0.5245 | 0.6857 | 0.5273 | 0.4665 | 0.584 |
| P-value(Senior diagnosis standalone vs. AI diagnosis(G+C)) | 0.1836 | 0.7726 | 0.1218 | 0.1284 | 0.5155 | 0.002 |
| P-value(Senior diagnosis standalone vs. AI diagnosis(G+C+E)) | 0.11 | 0.9261 | 0.0442 | 0.0376 | 0.5411 | 0.003 |
| P-value(Senior diagnosis standalone vs. AI diagnosis(G+C+E+M)) | 0.0236 | 0.0662 | 0.098 | 0.0696 | 0.0368 | <0.001 |
| P-value(Senior diagnosis standalone vs. Senior diagnosis with AI assistant) | 0.2852 | 0.3148 | 0.571 | 0.4906 | 0.2687 | 0.141 |
| P-value(Senior diagnosis standalone vs. Junior diagnosis with AI assistant) | 0.8267 | 0.6267 | 0.5023 | 0.5207 | 0.7653 | 0.667 |
| P-value(Junior diagnosis with AI assistant vs. AI diagnosis(G)) | 0.3569 | 0.1833 | 0.504 | 0.9586 | 0.1979 | 0.526 |
| P-value(Junior diagnosis with AI assistant vs. AI diagnosis(G+C)) | 0.0083 | 0.836 | <0.001 | <0.001 | 0.6298 | <0.001 |
| P-value(Junior diagnosis with AI assistant vs. AI diagnosis(G+C+E)) | 0.0099 | 0.7796 | <0.001 | <0.001 | 0.6535 | <0.001 |
| P-value(Junior diagnosis with AI assistant vs. AI diagnosis(G+C+E+M)) | <0.001 | 0.0657 | <0.001 | <0.001 | 0.0098 | <0.001 |
| P-value(Junior diagnosis with AI assistant vs. Senior diagnosis with AI assistant) | 0.0703 | 0.4893 | 0.0992 | 0.0656 | 0.2895 | 0.465 |
| P-value(Senior diagnosis with AI assistant vs. AI diagnosis(G)) | 0.0412 | 0.0813 | 0.1768 | 0.0831 | 0.0542 | 0.512 |
| P-value(Senior diagnosis with AI assistant vs. AI diagnosis(G+D)) | 0.8556 | 0.411 | 0.2084 | 0.2905 | 0.532 | 0.019 |
| P-value(Senior diagnosis with AI assistant vs. AI diagnosis(G+C+E)) | 0.4912 | 0.4646 | 0.0588 | 0.0597 | 0.6765 | 0.027 |
| P-value(Senior diagnosis with AI assistant vs. AI diagnosis(G+C+E+M)) | 0.1057 | 0.4457 | 0.1578 | 0.1313 | 0.3185 | 0.01 |

Statistical quantifications were demonstrated with 95% confidence interval.

Abbreviations: US, ultrasound; G, gray-scale ultrasound; C, color Doppler flow imaging; E, strain elastography; M, region of interest mask; AUC, area under the receiver operator characteristic curve; PPV, positive predictive value; NPV, negative predictive value.

The accuracy, sensitivity, specificity, PPV, NPV and AUC were statistically compared to those of junior radiologists in the first diagnosis without DL-assistance, respectively (*, P<0.05; **, P<0.001). ^†^P values for statistical significance(<0.05).
